# Time to first remission and associated factors in type 2 diabetes mellitus: a retrospective cohort study in Ethiopia

**DOI:** 10.1186/s12902-026-02346-3

**Published:** 2026-06-08

**Authors:** Ayitenew Agegn, Mengistu Abebe, Hailemariam Mamo Hassen

**Affiliations:** 1 Department of Statistics, College of Natural and Computational Sciences, Dire Dawa University, Dire Dawa, Ethiopia; 2 Department of Public Health, College of Medicine and Health Sciences, Dire Dawa University, P.O. Box: 1362, Dire Dawa, Ethiopia

**Keywords:** Time to remission, Type 2 diabetes mellitus, Survival analysis, Risk factors, Ethiopia

## Abstract

**Background:**

The prevalence of type 2 diabetes mellitus (T2DM) is rising in Ethiopia. Treatment for remission reduces further complications and the timing of these outcomes is influenced by various factors. However, there was a paucity of evidence in the Ethiopian context about these issues. This study examined the time to first remission and associated factors in T2DM patients treated at two referral hospitals in Eastern Ethiopia.

**Methods:**

A census-based retrospective cohort study was employed and analyzed secondary data from 1162 T2DM patients treated at two Hospitals between January 2017 and December 2021. Following descriptive analysis, Kaplan-Meier survival analysis was used to estimate the time to first remission, and Cox proportional hazards models were employed to identify factors associated with remission time.

**Results:**

Within a five-year follow-up period, 701 (60.33%) patients achieved first remission. Among those who achieved remission, 92.58% reached this milestone within the first 24 months of follow-up. Male gender, older age, family history of diabetes, comorbidities, increased cholesterol levels, and therapy at Dil-Chora Referral Hospital were all related to a longer duration of initial remission. Male gender was related to an 11.81% longer remission duration than females (HR = 0.8819, *p* = 0.006). Similarly, individuals with comorbidities had an 11.25% longer remission duration (HR = 0.8875, *p* < 0.001).

**Conclusion:**

A significant majority of T2DM remissions occur within a critical two-year window following treatment initiation, identifying this period as the golden opportunity’ for intensive clinical intervention. These findings underscore the necessity of implementing early, aggressive glycemic management and multidisciplinary care models to maximize remission potential. Future research should prioritize investigating the biological drivers of early treatment success and the long-term durability of remission in the Ethiopian context.

**Clinical trial number:**

Not applicable.

**Supplementary Information:**

The online version contains supplementary material available at 10.1186/s12902-026-02346-3.

## Introduction

While Type 2 Diabetes Mellitus (T2DM) has historically been managed as a chronic, progressive condition requiring lifelong medication, contemporary clinical evidence has shifted the focus toward metabolic remission [1]. T2DM remission—defined as achieving sub-diagnostic glycemic levels without active pharmacological intervention—is now recognized as a primary clinical endpoint that can arrest the progression of complications [2]. However, the physiological mechanisms and predictors of early remission are often studied in Western populations, leaving a critical gap in understanding how these trajectories manifest in sub-Saharan African cohorts [3].

Recent landmark studies, such as the Diabetes Remission Clinical Trial (DiRECT), have demonstrated that significant weight loss and intensive glycemic management can lead to T2DM reversal [4, 5] Despite these global advancements, there is limited evidence regarding the durability of remission in resource-limited settings. In the African context, unique factors such as dietary transitions, varying genetic predispositions, and differences in healthcare delivery systems may alter the expected time to first remission [3, 6]. Investigating these patterns is essential to validate international remission protocols within diverse clinical environments. In Ethiopia, the escalating burden of T2DM poses a substantial threat to a healthcare system already strained by a dual burden of infectious and chronic diseases [6]. This study is particularly significant for Ethiopia as it provides facility-based evidence from Eastern Ethiopia to inform national treatment guidelines. Identifying local prognostic factors for remission is a matter of economic and clinical urgency; it offers a pathway to reduce long-term dependency on costly medications and minimize the socioeconomic impact of diabetes-related disability. By tailoring management strategies to the Ethiopian clinical context, healthcare providers can prioritize metabolic recovery over lifelong disease maintenance.

T2DM can cause a variety of severe consequences [7, 8]. It can lead to serious health complications including heart disease, stroke, nerve damage, foot problems, vision loss, kidney disease, sexual problems, increased risk of miscarriage and stillbirth, weakened immune function, reduced respiratory function, and depression, in addition to common symptoms such as excessive thirst, frequent urination, and tiredness [7]. Furthermore, The present prevalence of T2DM and its projected rise create a huge public health challenge increasing diabetes-related healthcare expenditures [9, 10]. Healthcare expenditures for individuals with T2DM are projected to be significantly higher in 2040 compared to those without diabetes, with costs driven by factors such as medication costs, advanced age, high literacy, higher income, longer disease duration, multiple complications, and the use of combination therapies [7].

Healthy lifestyle choices and close collaboration with healthcare professionals help to manage or even reverse type 2 diabetes [11]. Hence, achieving and maintaining remission, defined as returning blood glucose levels to normal ranges without the need for diabetes medications [5, 12–18], is critical for avoiding these consequences and enhancing the quality of life for people with T2DM [19]. Remission is an important treatment objective since it lowers the risk of long-term problems while also improving overall health outcomes [1, 2, 8, 19–21].

Several factors can influence the time to initial remission in T2DM, including demographic parameters (age, gender), lifestyle factors (diet, physical activity), clinical factors (comorbidities, baseline blood glucose levels), and access to quality healthcare [11, 22–24]. Understanding the factors that influence the time to first remission is essential for developing effective treatment strategies, identifying high-risk individuals, and improving patient outcomes. Therefore, the objective of this retrospective cohort study was to examine the time to first remission and associated factors in T2DM patients treated at two referral hospitals in Eastern Ethiopia.

## Methods and materials

### Study design and data sources

The study was conducted at Hiwot Fana University Specialized Hospital (HFUSH) and Dil-Chora Referral Hospital (DCRH), the two major public healthcare facilities in Eastern Ethiopia. HFUSH is a specialized teaching hospital affiliated with Haramaya University, serving as a tertiary referral center for approximately 5 million people in the Harari Region and the surrounding Oromia and Somali regions. In contrast, DCRH is a regional referral hospital located in Dire Dawa, primarily serving the urban and peri-urban population of the city administration and neighboring zones. Both facilities follow the National Diabetes Management Guidelines established by the Ethiopian Federal Ministry of Health [25]. HFUSH operates as a high-volume academic center with integrated multidisciplinary clinics, whereas DCRH functions as a primary referral hub for chronic disease follow-up in the region.

The study approach was a retrospective cohort examining data from patients with Type 2 Diabetes Mellitus (T2DM) treated for a 60-month follow-up period from January 1, 2017, to December 30, 2021. Existing patient records from follow-up registries were used; any new data was not collected from participants. To ensure a well-defined inception cohort, the study specifically enrolled patients with newly diagnosed Type 2 Diabetes Mellitus (T2DM) who initiated their first course of treatment at Hiwot Fana Specialized University Hospital or Dil-Chora Referral Hospital during the study period.

The data-gathering procedure entailed each hospital’s T2DM wards or outpatient Departments, which included personal information, tracking cards, and clinical records. The data was collected using the standardized protocol for documenting clinical and laboratory measurements established by Ethiopia’s Federal Ministry of Health [26]. Approval of the study protocol and ethical clearance was obtained from the Dire Dawa University Institutional Review Board. Additionally, both hospitals’ medical directors provided letter of permission. The study population was patients aged 18 years and above with T2DM who began treatment at Dil-Chora Referral Hospital and Hiwot Fana University Special Hospital during the designated period.

### Sample size and sampling procedure

For this study, a formal sample size determination and sampling technique were not applied. Instead, a census-based approach was employed using secondary data from the medical records of two referral hospitals. All eligible records of patients with T2DM diagnosed and treated between January 2017 and December 2021 were reviewed. Records were selected for inclusion based on clearly defined criteria to ensure the relevance, accuracy, and consistency of the data with the study objectives, resulting in a final analyzed cohort of 1,162 patients.

Data selection was guided by strict inclusion and exclusion criteria. Patients were included if they were newly diagnosed with T2DM and had at least one year of follow-up data. Records with missing baseline biochemical parameters or incomplete follow-up histories were excluded to maintain the integrity of the time-to-event analysis. A total of 1,162 patients met the inclusion criteria: aged 18 or older, diagnosed with T2DM at the study hospitals, and possessing at least 12 months of follow-up data. Patients were excluded if they had incomplete baseline clinical data, were ‘transferred-in’ from other facilities after treatment initiation, or lacked documented follow-up outcomes, ensuring that the 1,162 included participants provided a robust and verifiable dataset for the time-to-remission analysis. Remission was defined as achieving a fasting blood sugar (FBS) level of 70–130 mg/dL on at least two consecutive follow-up visits (typically every 3 months) without medication.

### Outcome and explanatory variables

Remission was defined as achieving a fasting blood sugar (FBS) level of 70–130 mg/dL on at least two consecutive follow-up visits (typically every 3 months) without medication [27]. Biochemical parameters, including blood glucose levels, were analyzed as clinical prognostic indicators rather than primary explanatory variables. We acknowledge that while hyperglycemia is a diagnostic criterion for T2DM, baseline glucose levels and other biochemical shifts serve as essential markers of glycemic control and disease severity at the time of enrollment. These variables were included in the multivariable model to adjust for the baseline clinical state of the patient, allowing for a more nuanced analysis of how initial metabolic stability influences the time to first remission. The explanatory variables include sociodemographic characteristics (age, gender, place of residence (urban/rural), and family history of T2DM comorbidities, baseline laboratory values (fasting blood sugar, glucose, creatinine, hemoglobin, and cholesterol), and the treatment hospitals (Dil-Chora Referral Hospital vs. Hiwot Fana University Special Hospital).

### Definition of the event and outcome measurement

In this study, first remission was operationally defined as achieving a Fasting Blood Sugar (FBS) level between 70 and 130 mg/dL on at least two consecutive follow-up visits while not receiving any glucose-lowering medications. This definition aligns with the clinical guidelines for diabetes management in Ethiopia and international standards for assessing glycemic control without active pharmacological intervention. The event was recorded at the date of the first clinical assessment where these criteria were met. For patients who did not achieve remission by the end of the 60-month study period, data were censored at the date of their last documented follow-up or at the time of death or loss to follow-up. This rigorous timeline ensured that the survival functions accurately reflected the duration from treatment initiation to the physiological stabilization of the patient.

### Statistical analysis

Stata software was for data analysis. Descriptive analysis was used to summarize baseline characteristics of the study population (mean, standard deviation, and frequencies) and to describe of time to remission in the course of 60 months. For the survival analysis the Kaplan-Meier method, log-rank test, and Cox Proportional Hazards Model were used. The Kaplan-Meier method was employed to estimate the survival function (time to first remission) and generate survival curves. The log-rank test was used to compare survival curves between different patient groups (e.g., gender, presence of comorbidities, treatment hospital). The Cox Proportional Hazards Model was utilized to identify factors significantly associated with time to first remission. To identify candidate variables for the multivariable analysis, a bivariable (univariate) analysis was first conducted. Variables demonstrating a p-value < 0.25 in the bivariable analysis were considered for inclusion in the final Cox proportional hazards model. This more liberal screening threshold was chosen over the traditional 0.05 to ensure that potential confounders and variables that might become significant when adjusted for other factors were not prematurely excluded from the model. We estimated hazard ratios (HRs) and 95% confidence intervals (CIs) to assess the strength and direction of the association between each predictor variable and time to first remission.

## Results

### Study participants’ profile

The study included Patients with Type 2 Diabetes Mellitus (T2DM) who were 1162 in number treated at two referral hospitals in Eastern Ethiopia. The majority (56.97%) of patients received treatment at Hiwot Fana University Specialist Hospital. The mean age of the study participants was 37.52 years with a standard deviation of ± 14.451 years. The majority of the study population fell within 17 to 68 years age group, as detailed in the socio-demographic summary in Table 1. The cohort was composed of 52.93% females and 47.07% males and 66.01% of them had no family history of diabetes. Around two-thirds of the participants (66.27%) were from urban areas. In terms of other health conditions, 73.32% did not have complex conditions. In terms of cholesterol levels, 65.06% were normal, with 28.92% having elevated levels (Table 1). Table 1 shows that 423 patients (36.4%) from Hiwot Fana and 278 patients (23.92%) from Dil Chora experienced an “observed event,” representing those who achieved Type 2 Diabetes remission within sixty months. However, 239 patients (20.57%) from Hiwot Fana and 222 patients (19.11%) from Dil Chora were “censored,” indicating either no remission or incomplete follow-up (Table 1).

**Table 1:** Baseline demographic characteristics of patients with Type 2 Diabetes Mellitus

Characteristic variables	Category	Total cases (*N* = 1162)	*Total observed events (n = 701)*	*Censored (n = 461)*
		n (%)	n (%)	n (%)
Cases by hospital	Cases from Hiwot Fana	662(56.97)	*423(36.4)*	*239(20.57)*
	Cases from Dil Chora	500(43.03)	*278(23.92)*	*222(19.11)*
Gender	Female	615(52.93)	*397(34.16)*	*218(18.76)*
	Male	547(47.07)	*304(26.16)*	*243(20.91)*
Family history of diabetes mellitus	No	767(66.01)	*493(42.43)*	*274(23.58)*
	Yes	395(33.99)	*208(17.9)*	*187(16.09)*
Residence	Rural	392(33.73)	*211(18.16)*	*181(15.57)*
	Urban	770(66.27)	*490 (42.17)*	*280(24.1)*
Complicated disease	No	852(73.32)	*579 (49.83)*	*273(23.49)*
	Yes	310 (26.68)	*122(10.5)*	*188(16.18)*
Cholesterol level	Normal	756(65.06)	*511(43.98)*	*245(21.08)*
	Borderline High	76(6.54)	*25(2.15)*	*51(4.39)*
	High	336(28.92)	*175(15.06)*	*161(13.86)*

The mean fasting blood glucose level was 284.56 mg/dL, with a standard deviation of 86.85 mg/dL which shows the study participants had elevated blood sugar levels. Creatinine level on average was 1.3546 mg/dL, with a standard deviation of 0.86743 mg/dL, indicating reasonably normal renal function in the studied cohort. The mean hemoglobin level was 13.694 g/dL, with a standard deviation of 1.6017 g/dL, indicating relatively normal hemoglobin levels in the study population.

### Description of time to remission in months

**Fig. 1:**
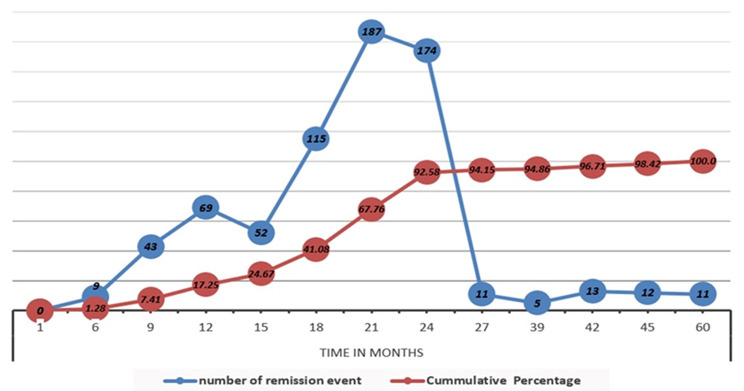
Table: Description of time to remission in months

The time to first remission for Type 2 Diabetes followed a dynamic pattern (Fig. 1). A substantial number of patients (92.58%) achieved remission within the first 24 months of treatment. However, the remission rate slowed down significantly after two years. Within 18 months, approximately 50% of patients achieved remission, indicating that this could be a significant milestone. This pattern emphasizes the necessity for continued monitoring and potential changes to treatment strategies, particularly for those who do not achieve remission within the first two years.

### Survival curve analysis

**Fig. 2:**
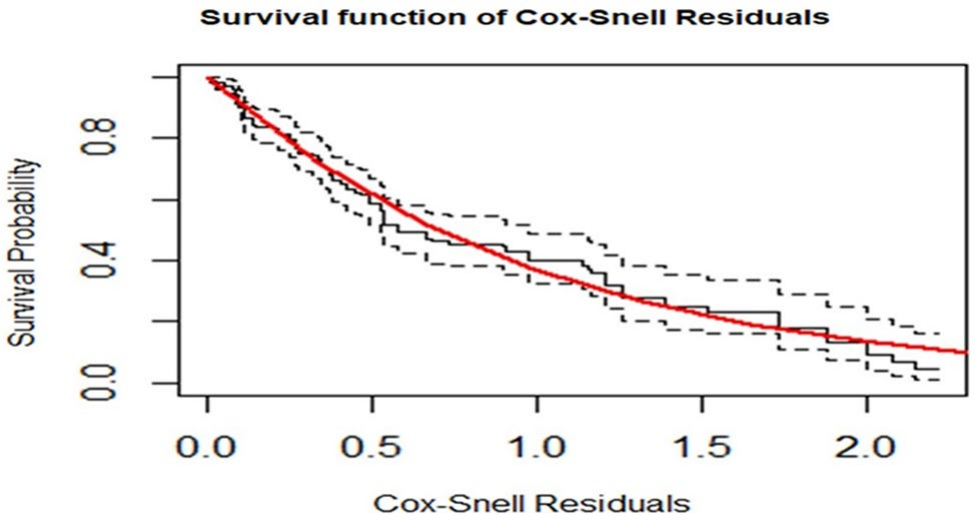
The validity of the proportional hazards model using the Cox-Snell residual

The proportional hazards assumption of the Cox model was tested with Cox-Snell residuals (Fig. 2). The Kaplan-Meier estimate of these residuals aligned with the expected exponential distribution closely suggesting a good fit of the model to the data. Further testing with Cox-Snell residuals validated the model’s adequacy, demonstrating that the survival process model accurately represents the observed data.

**Fig. 3:**
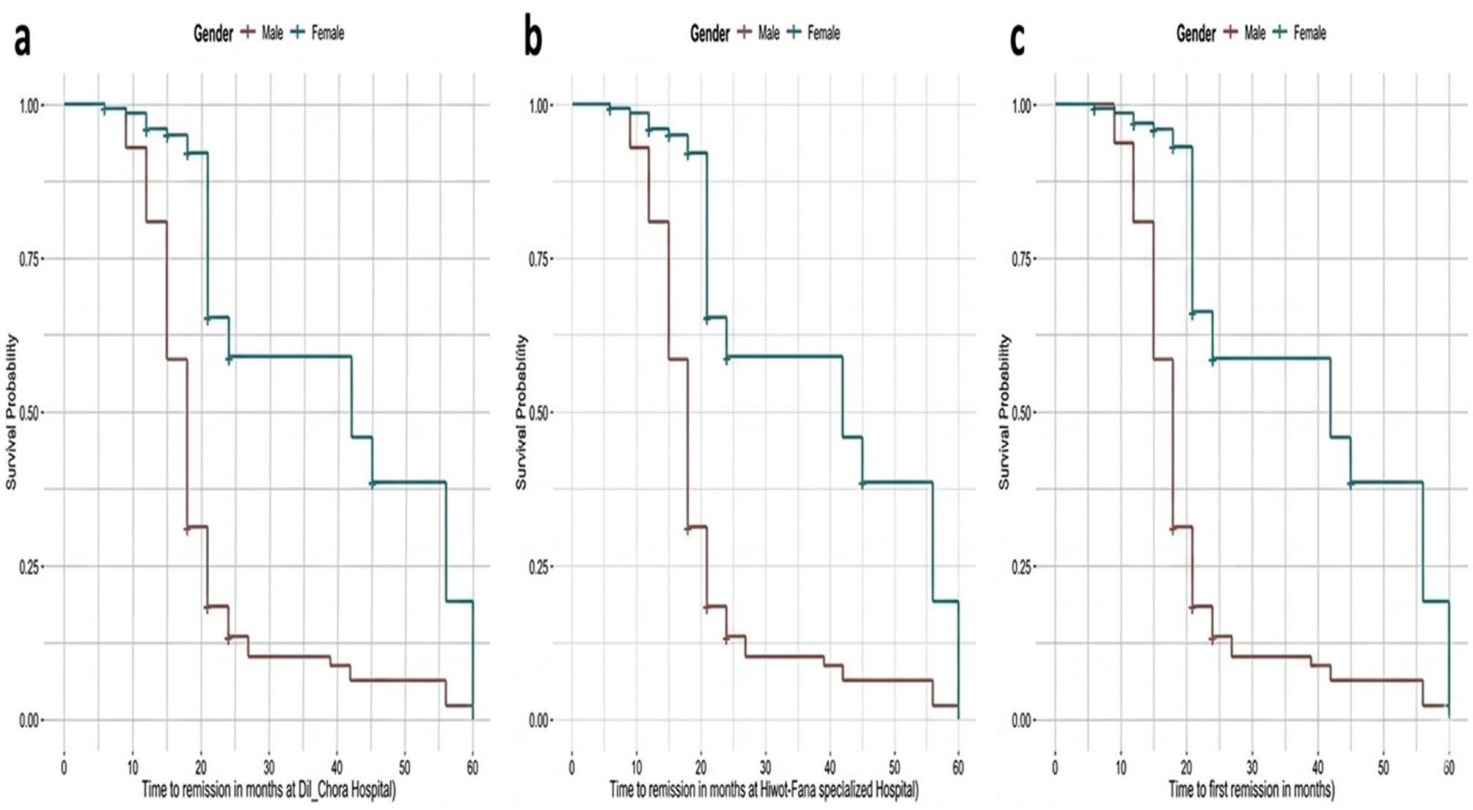
Estimate of kaplan-meier survivor function plot of T2DM patients by hospitals and gender

The overall Kaplan-Meier survival estimate for the time to first remission is illustrated in Fig. 3. The cumulative probability of achieving remission increased most significantly during the first 24 months of follow-up, after which the rate of new remission events began to plateau.

Male patients exhibited a longer duration to first remission compared to female patients, suggesting potential gender-based differences in disease progression or treatment response.

**Fig. 4:**
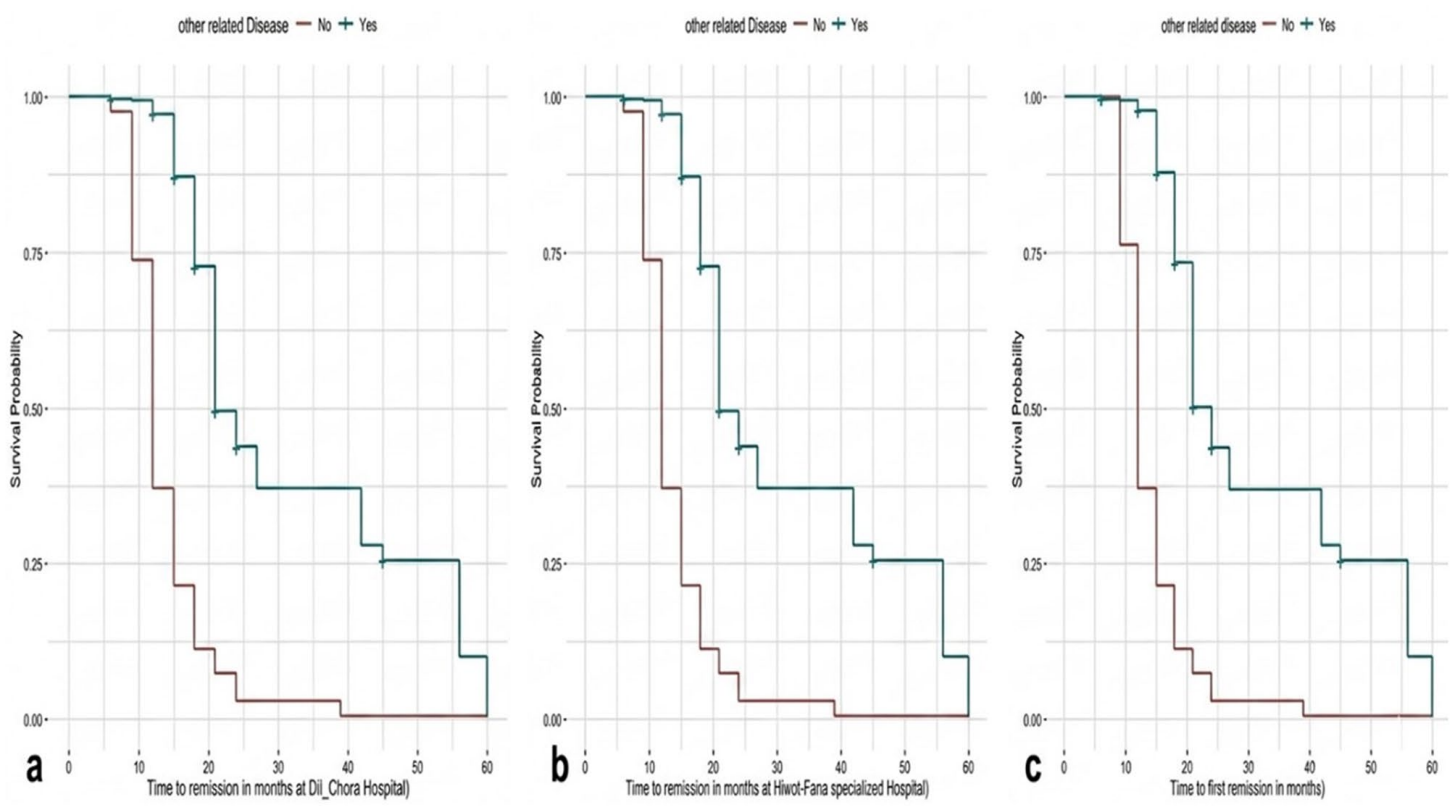
Estimate of kaplan-meier survivor function plot of T2DM patients by comorbidities

The Kaplan-Meier analysis provides a comprehensive overview of the transition to remission across the study population. The aggregate survival function (Fig. 4b) illustrates the overall tempo of recovery, with the steepest decline in non-remission probability occurring between 10 and 25 months. When stratified by facility (Fig. 4a), a distinct performance gap emerged; patients at Hiwot Fana Specialized University Hospital reached first remission significantly faster than those at Dil-Chora Referral Hospital, as evidenced by the notably lower cumulative survival curve for the former. Furthermore, the analysis of clinical determinants (Fig. 4c) revealed that comorbidity status significantly delayed recovery. Patients with comorbidities maintained a higher survival probability (representing a longer time to remission) compared to those without comorbidities. This disparity was particularly pronounced at Dil-Chora, where the presence of comorbidities appeared to exert a stronger inhibitory influence on remission timelines than at Hiwot Fana.

**Fig. 5:**
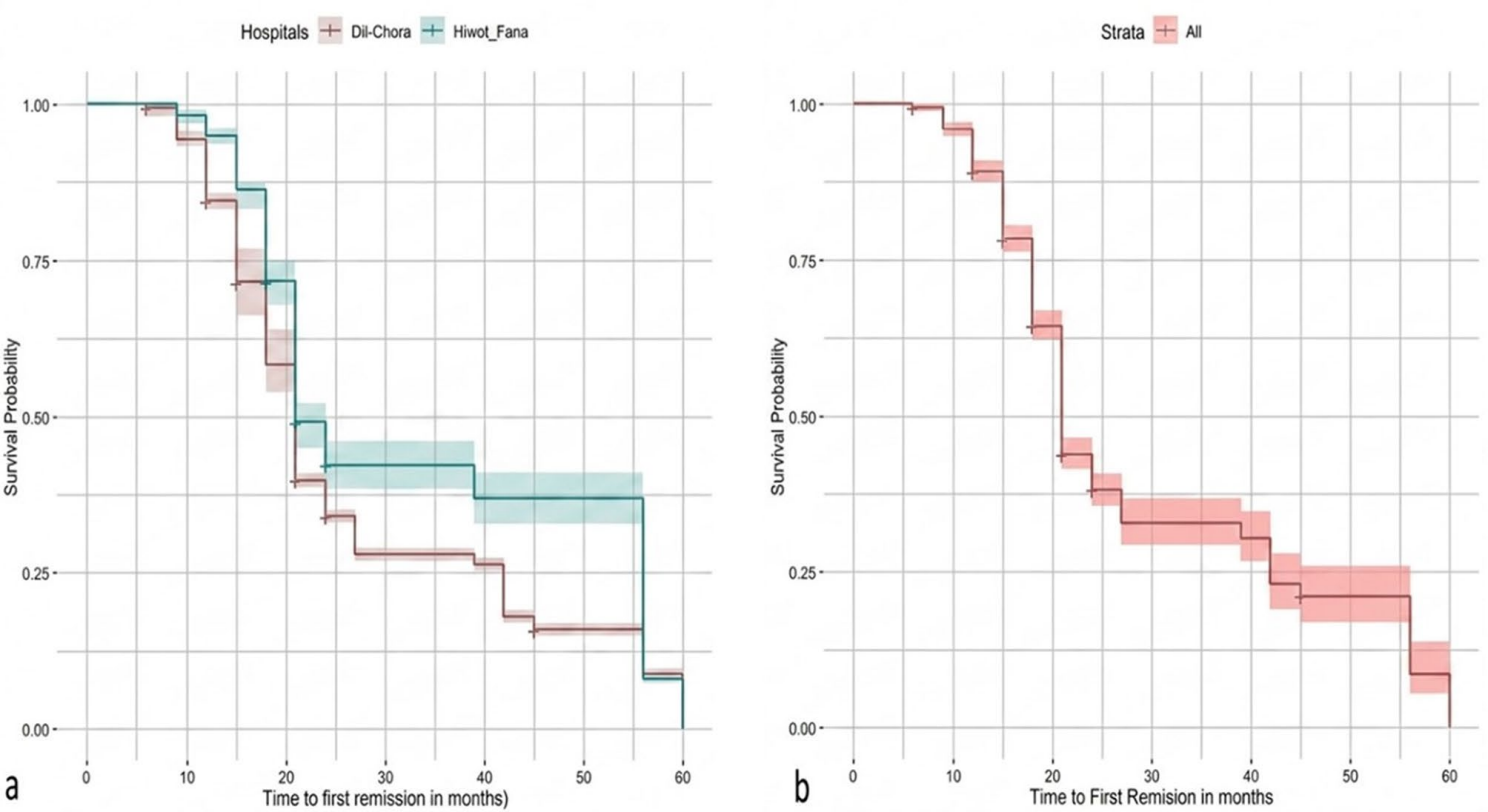
Comparison of time to first remission between hospitals for type 2 diabetes patients

The analysis of remission timing across the study sites is presented in Fig. 5. The stratified Kaplan-Meier curves (Fig. 5a) demonstrate a comparable trend in the time to first remission between the two facilities. Although Dil-Chora Referral Hospital showed a slightly earlier decline in the probability of remaining in a non-remission state during the initial 20 months, the overall difference between the institutions was not statistically significant (Log-rank *p* ≥ 0.05). This suggests that the clinical progression toward remission is consistent across both specialized settings. Furthermore, the aggregate survival function for the entire cohort (Fig. 5b) illustrates the overall pace of recovery, with the shaded area representing the 95% confidence interval for the cumulative remission probability.

### Factors influencing time to first remission in Type 2 diabetes mellitus

The multivariate Cox proportional hazards model identified characteristics that significantly influenced the time to first remission in Type 2 Diabetes patients (Table 2). Male gender was related to an 11.81% longer remission duration than females (HR = 0.8819, *p* = 0.006). Similarly, individuals with comorbidities experienced 11.25% shorter remission time (HR = 0.8875, *p* < 0.001).

**Table 2:** Factors associated with time to first remission in Type 2 diabetes mellitus

Covariate	Estimate	Std. Err	aHR	95%CI	*P*-Value
Age	-0.1419	0.0703	0.8676	(0.7560,0.9959)	0.04358
Hospital (Ref = Dil Chora)					
Hiwot Fana	0.24	0.1123	1.27	(1.0201,1.5842)	0.0436
Family History (Ref = No)					
Yes	-0.1539	0.0276	0.8574	(0.8122,0.9051)	< 0.001
Gender (Ref=Female)					
Male	-0.1256	0.0461	0.8819	(0.8058,0.9654)	0.006
Patients With Comorbidities (Ref = No)					
Yes	-0.1193	0.0867	0.8875	(0.7488,1.0519)	< 0.001
Residence(Ref=Rural)					
Urban	0.4975	0.2929	1.6446	(0.9663,2.9200)	0.0894
Hemoglobin	0.6482	0.0891	1.9121	(1.6057,2.2769)	< 0.001
Cholesterol Level (Ref = Normal)					
Borderline High	-0.986	1.1321	0.3731	(0.0406,2.4312)	0.873
High	-0.5418	0.1019	0.5817	(0.4764,0.7103)	< 0.001

Patients treated at Hiwot Fana University Specialist Hospital had a 27% longer time to remission compared to those at Dil Chora Referral Hospital (HR = 1.27, *p* = 0.0436). Older age (HR = 0.8676, *p* = 0.04358) and a family history of diabetes (HR = 0.8574, *p* < 0.0001) were associated with shorter remission periods. Elevated cholesterol levels were linked to a 41.83% lower probability of achieving first remission (HR = 0.5817, *p* < 0.001). Additionally, patients with a family history of diabetes had a 14.26% lower recovery rate compared to those without a family history (HR = 0.8574, p-value < 0.001).

## Discussion

This retrospective cohort study investigated the time to first remission and associated factors in a cohort of 1162 Type 2 Diabetes Mellitus (T2DM) patients treated at two referral hospitals in Eastern Ethiopia. Our findings revealed a dynamic pattern of remission, with a substantial proportion of patients achieving remission within the first two years of treatment, followed by a significant decline in the remission rate. Gender, comorbidities, age, family history of diabetes, cholesterol levels, and the treating hospital had a substantial impact on the time to first remission, emphasizing the importance of a nuanced and tailored approach to T2DM care in this study setting.

Contrary to the traditional understanding of age as a risk factor for disease progression, our findings indicated that older age was associated with a shorter time to first remission [28]. This apparent paradox may be explained by higher levels of treatment adherence and health-seeking behavior often observed in older patient populations within this region. Younger patients may face unique barriers, such as occupational demands or lower perceived risk, leading to inconsistent follow-up [29]. Furthermore, this trend may reflect a ‘survivor bias,’ where older individuals in the cohort represent a subset with fewer life-threatening comorbidities or more stable metabolic profiles, allowing for a quicker response to therapeutic interventions compared to younger counterparts who may present with more aggressive or early-onset forms of the disease [28].

One of the key findings was the association between male gender and a longer time to first remission. This observation, which is consistent with certain earlier investigations [11, 22–24], necessitates further investigation to determine the underlying mechanisms. The possible explanations could be gender-specific differences in illness etiology, therapeutic response, and adherence to treatment regimens [8, 16, 24, 30, 31]. It implies that gender may influence illness progression, therapy response, and treatment regimen adherence. Type 2 Diabetes Mellitus (T2DM) patients with comorbidities had a significantly longer duration to first remission than those without comorbidities. The large impact of comorbidities on the time to first remission is consistent with previous research findings [32, 33].

The presence of other chronic illnesses might complicate diabetes management and increase the risk of complications, thereby prolonging the time required to achieve and sustain remission [34]. Our findings also highlight the influence of age and family history of diabetes on remission time. Older age and a positive family member history of diabetes were related to shorter remission times, most likely due to the cumulative influence of the disease and genetic susceptibility, respectively [2, 8, 10, 19, 32, 34–36].

Another finding of the present study was a substantial difference in remission time between patients treated in the two facilities patients at Hiwot Fana University Specialist Hospital had remission much faster than those at Dil-Chora Referral Hospital. Variations in access to specialized care, disparities in treatment protocols and adherence, resource availability, and variations in the patient groups treated by each hospital could all contribute to this disparity [37, 38]. Further investigation is warranted to identify the specific factors contributing to this disparity and to explore strategies for improving outcomes at Dil-Chora Referral Hospital. The observed discrepancy in remission timelines between the two facilities may be rooted in variations in clinical protocols and resource allocation. At Hiwot Fana Specialized University Hospital, the integration of academic research with clinical practice often facilitates a multidisciplinary team (MDT) approach, where endocrinologists, nutritionists, and specialized nurses conduct joint reviews—a protocol less consistently implemented at Dil-Chora Referral Hospital due to higher patient-to-specialist ratios. Furthermore, Hiwot Fana utilizes a structured ‘Early Intensive Glycemic Control’ protocol, which emphasizes aggressive initial management and bi-weekly follow-ups during the first three months. In contrast, Dil-Chora follows the standard regional referral guidelines which, while robust, may lack the same frequency of specialized laboratory monitoring and patient education intensity found in a university-affiliated setting.

The study highlights the evidence for tailored therapy techniques addressing individual patient characteristics while improving remission rates and long-term treatment outcomes. However, this study has limitations that must be noted. As a retrospective study, it has the possibility of data inconsistencies and missing information. The study only included patients from two hospitals, so it may not be representative of the entire T2DM community in Eastern Ethiopia. The relatively short follow-up time and censored nature of the study may have reduced the ability to observe long-term effects and estimate the durability of remission.

## Conclusion

This study provides valuable insights into the time to first remission and its associated factors among T2DM patients in Eastern Ethiopia. The findings underscore the importance of considering a range of factors, including gender, comorbidities, age, family history, and the treating institution when managing T2DM patients. These findings can inform the development of more targeted and personalized treatment strategies that address individual patient characteristics to improve remission rates and long-term outcomes for T2DM patients. The observed differences in remission time between hospitals emphasize the importance of improving access to quality care and ensuring equitable treatment outcomes across different healthcare settings. Further research is warranted to explore factors associated with early versus delayed remission and the underlying mechanisms for optimizing treatment strategies.

## Supplementary Information

Below is the link to the electronic supplementary material.

Supplementary Material 1

## Data Availability

The data is available at the correspondence author as per request.

## Abbreviations

aHR: Adjusted Hazard Ratio
BMC: BioMed Central
CI: Confidence Interval
DCRH: Dil-Chora Referral Hospital
FBS: Fasting Blood Sugar
HFUSH: Hiwot Fana University Specialized Hospital
HR: Hazard Ratio
MDT: Multidisciplinary Team
NHS: National Health Service
T2DM: Type 2 Diabetes Mellitus
