# Supplementary material for: Time to first remission and associated factors in type 2 diabetes mellitus: a retrospective cohort study in Ethiopia
Source: BMC Endocr Disord. 2026 Jun 8;26:232. doi: 10.1186/s12902-026-02346-3 (PMC13471171; doi:10.1186/s12902-026-02346-3)
Supplement: Supplementary file 1 [file 12902_2026_2346_MOESM1_ESM.pdf]

Additional File 1: STROBE Checklist

	Item No.	Recommendation	Page No.	Relevant text from manuscript
Title and abstract	1	(*a*) Indicate the study's design with a commonly used term in the title or the abstract	1	(*a*) Time to First Remission and Associated Factors in Type 2 Diabetes Mellitus: A Retrospective Cohort Study in Ethiopia
		(*b*) Provide in the abstract an informative and balanced summary of what was done and what was found	1	(*b*) A census-based retrospective cohort study was employed and analyzed secondary data from 1162 T2DM patients treated at two Hospitals between January 2017 and December 2021.
Introduction				
Background/rationale	2	Explain the scientific background and rationale for the investigation being reported	Page 2 (Introduction section)	While Type 2 Diabetes Mellitus (T2DM) has historically been managed as a chronic, progressive condition requiring lifelong medication, contemporary clinical evidence has shifted the focus toward metabolic remission [1]. T2DM remission—defined as achieving sub-diagnostic glycemic levels without active pharmacological intervention—is now recognized as a primary clinical endpoint that can arrest the progression of complications[2]. However, the physiological mechanisms and predictors of early remission are often studied in Western populations, leaving a critical gap in understanding how these trajectories manifest in sub-Saharan African cohorts[3]. Recent landmark studies, such as the Diabetes Remission Clinical Trial (DiRECT), have demonstrated that significant weight loss and intensive glycemic management can lead to T2DM reversal[4], [5] Despite these global advancements, there is limited evidence regarding the durability of remission in resource-limited settings. In the African context, unique factors such as dietary transitions, varying genetic predispositions, and differences in healthcare delivery systems may alter the expected time to first remission [3], [6] Investigating these patterns is essential to validate international remission protocols within diverse clinical environments. In Ethiopia, the escalating burden of T2DM poses a substantial threat to a healthcare system already strained by a dual burden of infectious and chronic diseases[6]. This study is particularly significant for Ethiopia as it provides facility-based evidence from Eastern Ethiopia to inform national treatment guidelines. Identifying local prognostic factors for remission is a matter of economic and clinical urgency; it offers a pathway to reduce long-term dependency on costly medications and minimize the socioeconomic impact of diabetes-related disability. By tailoring management strategies to the Ethiopian clinical context, healthcare providers can prioritize metabolic recovery over lifelong disease maintenance.
Objectives	3	State specific objectives, including any prespecified hypotheses	Page 1 (Abstract) and Page 2/3 (End of Introduction)	This study examined the time to first remission and associated factors in T2DM patients treated at two referral hospitals in Eastern Ethiopia.

Methods				
Study design	4	Present key elements of study design early in the paper	Pages 1–2	The study approach was a retrospective cohort examining data from patients with Type 2 Diabetes Mellitus (T2DM) treated for a 60-month follow-up period from January 1, 2017, to December 30, 2021. Existing patient records from follow-up registries were used; any new data was not collected from participants. To ensure a well-defined inception cohort, the study specifically enrolled patients with newly diagnosed Type 2 Diabetes Mellitus (T2DM) who initiated their first course of treatment at Hiwot Fana Specialized University Hospital or Dil-Chora Referral Hospital during the study period.
Setting	5	Describe the setting, locations, and relevant dates, including periods of recruitment, exposure, follow-up, and data collection	Methods and Materials *(Under Study Design and data sources and Sample Size and Sampling Procedure*	The study was conducted at Hiwot Fana University Specialized Hospital (HFUSH) and Dil-Chora Referral Hospital (DCRH), the two major public healthcare facilities in Eastern Ethiopia. HFUSH is a specialized teaching hospital affiliated with Haramaya University, serving as a tertiary referral center for approximately 5 million people in the Harari Region and the surrounding Oromia and Somali regions. In contrast, DCRH is a regional referral hospital located in Dire Dawa, primarily serving the urban and peri-urban population of the city administration and neighboring zones. The study approach was a retrospective cohort examining data from patients with Type 2 Diabetes Mellitus (T2DM) treated for a 60-month follow-up period from January 1, 2017, to December 30, 2021... To ensure a well-defined inception cohort, the study specifically enrolled patients with newly diagnosed Type 2 Diabetes Mellitus (T2DM) who initiated their first course of treatment at Hiwot Fana Specialized University Hospital or Dil-Chora Referral Hospital during the study period. All eligible records of patients with T2DM diagnosed and treated between January 2017 and December 2021 were reviewed... Patients were included if they were newly diagnosed with T2DM and had at least one year of follow-up data.The data-gathering procedure entailed each hospital's T2DM wards or outpatient Departments, which included personal information, tracking cards, and clinical records. The data was collected using the standardized protocol for documenting clinical and laboratory measurements established by Ethiopia's Federal Ministry of Health.
Participants	6	(*a*) *Cohort study*—Give the eligibility criteria, and the sources and methods of selection of participants. Describe methods of follow-up	Within the Methods and Materials section under the sub-headings Study Design and data sources, Sample Size and Sampling Procedure, and Definition of the	*1. Eligibility Criteria (Inclusion and Exclusion Criteria)*<br>*The study population was patients aged 18 years and above with T2DM who began treatment at Dil-Chora Referral Hospital and Hiwot Fana University Special Hospital during the designated period.*<br>*Data selection was guided by strict inclusion and exclusion criteria. Patients were included if they were newly diagnosed with T2DM and had at least one year of follow-up data. Records with missing baseline biochemical parameters or incomplete follow-up histories were excluded to maintain the integrity of the time-to-event analysis.*<br>*A total of 1,162 patients met the inclusion criteria: aged 18 or older, diagnosed with T2DM at the study hospitals, and possessing at least 12 months of follow-up data. Patients were excluded if they had incomplete baseline clinical data, were 'transferred-in' from other facilities after treatment initiation, or lacked documented follow-up outcomes, ensuring that the 1,162 included participants provided a robust and verifiable dataset for the time-to-remission analysis.*<br>*2. Sources and Methods of Selection of Participants*<br>*The study was conducted at Hiwot Fana University Specialized Hospital (HFUSH) and Dil-Chora Referral Hospital (DCRH), the two major public healthcare facilities in Eastern Ethiopia.*<br>*To ensure a well-defined inception cohort, the study specifically enrolled patients with newly diagnosed Type 2 Diabetes Mellitus (T2DM) who initiated their first course of treatment at Hiwot Fana Specialized University Hospital or Dil-Chora Referral Hospital during the study period.*<br>*The data-gathering procedure entailed each hospital's T2DM wards or outpatient Departments, which included personal information, tracking cards, and clinical records.*

			Event and Outcome Measurement	*For this study, a formal sample size determination and sampling technique were not applied. Instead, a census-based approach was employed using secondary data from the medical records of two referral hospitals. All eligible records of patients with T2DM diagnosed and treated between January 2017 and December 2021 were reviewed.*<br>*3. Methods of Follow-up*<br>*The study approach was a retrospective cohort examining data from patients with Type 2 Diabetes Mellitus (T2DM) treated for a 60-month follow-up period from January 1, 2017, to December 30, 2021. Existing patient records from follow-up registries were used; any new data was not collected from participants.*<br>*Remission was defined as achieving a fasting blood sugar (FBS) level of 70–130 mg/dL on at least two consecutive follow-up visits (typically every 3 months) without medication.*<br>*For patients who did not achieve remission by the end of the 60-month study period, data were censored at the date of their last documented follow-up or at the time of death or loss to follow-up. This rigorous timeline ensured that the survival functions accurately reflected the duration from treatment initiation to the physiological stabilization of the patient.*
Variables	7	Clearly define all outcomes, exposures, predictors, potential confounders, and effect modifiers. Give diagnostic criteria, if applicable	Page 4 & Page 5 (corresponds to sections 2.3, 2.4, and 3.1 of the text)	Outcomes (Remission) & Diagnostic Criteria: Remission was defined as achieving a fasting blood sugar (FBS) level of 70–130 mg/dL on at least two consecutive follow-up visits (typically every 3 months) without medication. In this study, first remission was operationally defined as achieving a Fasting Blood Sugar (FBS) level between 70–130 mg/dL on at least two consecutive follow-up visits while not receiving any glucose-lowering medications. This definition aligns with the clinical guidelines for diabetes management in Ethiopia and international standards for assessing glycemic control without active pharmacological intervention. The event was recorded at the date of the first clinical assessment where these criteria were met.<br>Exposures, Predictors, and Potential Confounders: The explanatory variables include sociodemographic characteristics (age, gender, place of residence (urban/rural), and family history of T2DM comorbidities, baseline laboratory values (fasting blood sugar, glucose, creatinine, hemoglobin, and cholesterol), and the treatment hospitals (Dil-Chora Referral Hospital vs. Hiwot Fana University Special Hospital). Biochemical parameters, including blood glucose levels, were analyzed as clinical prognostic indicators rather than primary explanatory variables... These variables were included in the multivariable model to adjust for the baseline clinical state of the patient, allowing for a more nuanced analysis of how initial metabolic stability influences the time to first remission.<br>Participant Profiles & Characteristics: The mean age of the study participants was 37.52 years with a standard deviation of ± 14.451 years... The cohort was composed of 52.93% females and 47.07% males and 66.01% of them had no family history of diabetes. Around two-thirds of the participants (66.27%) were from urban areas. In terms of other health conditions, 73.32% did not have complex conditions. In terms of cholesterol levels, 65.06% were normal, with 28.92% having elevated levels

Data sources/ measurement	8*	For each variable of interest, give sources of data and details of methods of assessment (measurement). Describe comparability of assessment methods if there is more than one group	Page 4 (from the Methods and Materials section under the subheadings *Study Design and data sources* and *Outcome and explanatory variables*)	The data-gathering procedure entailed each hospital's T2DM wards or outpatient Departments, which included personal information, tracking cards, and clinical records. The data was collected using the standardized protocol for documenting clinical and laboratory measurements established by Ethiopia's Federal Ministry of Health.<br>Remission was defined as achieving a fasting blood sugar (FBS) level of 70–130 mg/dL on at least two consecutive follow-up visits (typically every 3 months) without medication. Biochemical parameters, including blood glucose levels, were analyzed as clinical prognostic indicators rather than primary explanatory variables.<br>The explanatory variables include sociodemographic characteristics (age, gender, place of residence (urban/rural), and family history of T2DM comorbidities, baseline laboratory values (fasting blood sugar, glucose, creatinine, hemoglobin, and cholesterol), and the treatment hospitals (Dil-Chora Referral Hospital vs. Hiwot Fana University Special Hospital).
Bias	9	Describe any efforts to address potential sources of bias	Page No.: *(Please confirm your final formatted manuscript page number, usually around Page 5 or 6 under Methods)*	To ensure a well-defined inception cohort, the study specifically enrolled patients with newly diagnosed Type 2 Diabetes Mellitus (T2DM) who initiated their first course of treatment at Hiwot Fana Specialized University Hospital or Dil-Chora Referral Hospital during the study period. *(And from your discussion regarding potential bias)*: Furthermore, this trend may reflect a 'survivor bias,' where older individuals in the cohort represent a subset with fewer life-threatening comorbidities or more stable metabolic profiles... As a retrospective study, it has the possibility of data inconsistencies and missing information.
Study size	10	Explain how the study size was arrived at	Page No.: *Not formally structured by page numbering in the source text, but located*	For this study, a formal sample size determination and sampling technique were not applied. Instead, a census-based approach was employed using secondary data from the medical records of two referral hospitals. All eligible records of patients with T2DM diagnosed and treated between January 2017 and December 2021 were reviewed. Records were selected for inclusion based on clearly defined criteria to ensure the relevance, accuracy, and consistency of the data with the study objectives, resulting in a final analysed cohort of 1,162 patients.

			directly under the Sample Size and Sampling Procedure sub-section within the Methods and Materials section.	

Continued on next page

Quantitative variables	11	Explain how quantitative variables were handled in the analyses. If applicable, describe which groupings were chosen and why	Page 4-5	To identify candidate variables for the multivariable analysis, a bivariable (univariate) analysis was first conducted. Variables demonstrating a p-value < 0.25 in the bivariable analysis were considered for inclusion in the final Cox proportional hazards model.<br>The mean age of the study participants was 37.52 years with a standard deviation of ± 14.451 years. The majority of the study population fell within 17 to 68 years age group, as detailed in the socio-demographic summary in Table 1.<br>In terms of cholesterol levels, 65.06% were normal, with 28.92% having elevated levels (Table 1). The mean fasting blood glucose level was 284.56 mg/dL, with a standard deviation of 86.85 mg/dL... Creatinine level on average was 1.3546 mg/dL, with a standard deviation of 0.86743 mg/dL... The mean hemoglobin level was 13.694 g/dL, with a standard deviation of 1.6017 g/dL...
Statistical methods	12	(*a*) Describe all statistical methods, including those used to control for confounding		Data entry, cleaning, and statistical analyses were performed using Stata software. Descriptive statistics (means, standard deviations for continuous variables; frequencies and percentages for categorical variables) were calculated to summarize baseline socio-demographic and clinical characteristics of the cohort. Time-to-event (first remission) was estimated using the non-parametric Kaplan-Meier survival method. To identify independent predictors and control for confounding factors, Cox proportional hazards regression models were constructed. Potential confounding variables—including age, gender, hospital site, family history of diabetes, residence, baseline hemoglobin, and baseline cholesterol levels—were prioritized and adjusted for concurrently within the multivariable Cox proportional hazards model to estimate Adjusted Hazard Ratios (aHR) and their corresponding 95% Confidence Intervals (CIs).
		(*b*) Describe any methods used to examine subgroups and interactions		The log-rank test was used to compare survival curves between different patient groups (e.g., gender, comorbidities, hospital).
		(*c*) Explain how missing data were addressed		Records with missing baseline biochemical parameters or incomplete follow-up histories were excluded...
		(*d*) *Cohort study*—If applicable, explain how loss to follow-up was addressed		Data were censored at the date of their last documented follow-up or at the time of death or loss to follow-up
		(*e*) Describe any sensitivity analyses		The proportional hazards assumption of the Cox model was tested with Cox-Snell residuals... suggesting a good fit.
**Results**				
Participants	13*	(a) Report numbers of individuals at each stage of study—eg numbers potentially eligible, examined for eligibility, confirmed eligible, included in the study, completing follow-up, and analysed		*Report numbers of individuals at each stage of study—eg numbers potentially eligible, examined for eligibility, confirmed eligible, included in the study, completing follow-up, and analysed.* Note: It also suggests giving reasons for non-participation at each stage and considering the use of a flow diagram.
		(b) Give reasons for non-participation at each stage		1. Statement for Exclusions at the Initial Screening Stage<br>Out of the total registry pool assessed between January 2017 and December 2021, secondary clinical records were excluded if they did not constitute part of a newly diagnosed inception cohort. Reasons for non-

				participation at this stage included a prior diagnosis of diabetes documented outside the predefined study enrolment window or clinical classification with a diagnosis other than Type 2 Diabetes Mellitus (T2DM) (such as type 1 or gestational diabetes).<br>2. Statement for Exclusions Due to Incomplete Clinical Records<br>A significant number of clinical entries were excluded from the final sample selection due to missing data essential for establishing follow-up time-to-event timelines. Specifically, records with missing baseline biochemical parameters—including initial fasting blood sugar measurements—or incomplete follow-up clinical tracking registries were excluded to preserve the accuracy and analytical integrity of the survival analysis.<br>3. Statement for Exclusions Due to Care Pathways or Follow-Up Violations<br>To minimize tracking bias, patients were systematically excluded if they were 'transferred-in' from other outside facilities after their first course of treatment had already initiated, as their exact treatment baseline could not be verified. Furthermore, patients who lacked a documented clinical follow-up outcome or had less than the mandated minimum of 12 months of consecutive tracking data within the designated hospital registries were categorized as non-participants and excluded from the analysis, resulting in a finalized sample of 1,162 patients.
Descriptive data	14*	(a) Give characteristics of study participants (eg demographic, clinical, social) and information on exposures and potential confounders	Results Section: Study participants' profile subsection	Detailed under Results: Study participants' profile, tracking Age, Gender, Residence, Family History, Hospital site, Comorbidities, and Baseline FBG, Creatinine, Hemoglobin, and Cholesterol levels. Cross-referenced to Table 1.
		(b) Indicate number of participants with missing data for each variable of interest	Sample Size and Sampling Procedure	descriptive completeness, a rigorous data pre-clearing protocol was applied during the registry extraction phase. Secondary clinical records presenting missing information for any of the main clinical or biochemical variables of interest (such as baseline fasting blood sugar, cholesterol levels, or tracking timelines) were systematically excluded from the analytical sample selection. Consequently, the finalized census cohort of 1,162 patients represents complete data profiles
		(c) *Cohort study*—Summarise follow-up time	Results section	The study captured clinical observation across a maximum tracking timeline of 60 months from Patients at Hiwot Fana University Specialized Hospital (HFUSH) and patients followed at Dil-Chora Referral Hospital (DCRH).
Outcome data	15*	*Cohort study*—Report numbers of outcome events or summary measures over time	Results section	Over the 60-month administrative follow-up period, a total of 701 (60.33%) patients experienced the primary outcome event of first metabolic remission. The incidence of remission events demonstrated a highly dynamic distribution over time. During the initial phases of treatment, remission events accumulated progressively, with 9 patients (1.28%) achieving remission by month 6, 43 patients (7.41%) by month 9, and 69 patients (17.25%) by month 12. A distinct acceleration in event frequency was observed between 15 and 24 months, peaking significantly at month 21 with 187 documented remission events (67.76% cumulative) and continuing at month 24 with 174 events, bringing the cumulative proportion of remissions within the first two years to 92.58% ($n = 649$ out of 701 events). Beyond this 24-month threshold, the rate of outcome events slowed down significantly, with minor distributions recorded across the remaining tracking timeline: 11 events at month 27, 5 at month 39, 13 at month 42, 12 at month 45, and a final 11 events reaching remission at the 60-month administrative censoring marker.

Main results	16	(*a*) Give unadjusted estimates and, if applicable, confounder-adjusted estimates and their precision (eg, 95% confidence interval). Make clear which confounders were adjusted for and why they were included	Results section	To evaluate predictors of time to first remission, bivariable Cox proportional hazards modeling was first executed to derive unadjusted estimates for each covariate. Confounder selection for the final multivariable Cox proportional hazards model was guided by a liberal screening threshold ($p < 0.25$) during bivariable analysis to ensure that potential suppressors, baseline clinical state indicators, and clinically relevant demographic parameters were not prematurely excluded.<br>Accordingly, the final multivariable model simultaneously adjusted for age, treating hospital, family history of diabetes, gender, comorbidity status, place of residence, baseline hemoglobin, and baseline cholesterol levels. These variables were included to control for disparities in health-seeking behavior (age, residence), differences in institutional care protocols (treating hospital), genetic predisposition (family history), biological/metabolic profiles (gender, hemoglobin, cholesterol), and clinical complexity (comorbidities).<br>After adjusting for these confounders, male gender (Adjusted Hazard Ratio [aHR] = 0.8819; 95% CI: 0.8058, 0.9654; $p = 0.006$) and the presence of comorbidities (aHR = 0.8875; 95% CI: 0.7488, 1.0519; $p < 0.001$) remained independently and significantly associated with a lower rate of achieving first remission (longer time to remission). Conversely, receiving care at Hiwot Fana University Specialist Hospital was associated with a 27% higher rate of achieving timely remission compared to Dil-Chora Referral Hospital (aHR = 1.27; 95% CI: 1.0201, 1.5842; $p = 0.0436$).
		(*b*) Report category boundaries when continuous variables were categorized	Results section	For analytical purposes, several continuous clinical and metabolic parameters were categorized based on established standard clinical diagnostic guidelines:<br>**Cholesterol Level:** Categorized into three distinct clinical strata according to the National Cholesterol Education Program (NCEP) Adult Treatment Panel III (ATP III) guidelines: *Normal* (< 200 mg/dL), *Borderline High* (200–239 mg/dL), and *High* (≥ 240 mg/dL).<br>**Hemoglobin (Hb) Level / Anemia Status:** Modeled as a continuous baseline predictor to preserve statistical power. However, clinical categorization for baseline physiological descriptions followed World Health Organization (WHO) thresholds, defining normal clinical boundaries as Hb ≥ 13.0 g/dL for adult males and Hb ≥ 12.0 g/dL for non-pregnant adult females.<br>**Age:** Maintained as a continuous variable in the Cox proportional hazards regression model to evaluate the linear hazard scale increment per added year of life, spanning a baseline participant range from 18 to 85 years.
		(*c*) If relevant, consider translating estimates of relative risk into absolute risk for a meaningful time period	Results section	Although the primary regression analysis utilizes Cox proportional hazards models to report relative measures (adjusted Hazard Ratios), absolute risk and event probabilities are fully integrated over clinically meaningful time periods. Specifically, the manuscript reports an overall absolute 5-year remission rate of 60.33% (701/1,162 patients). Stratified absolute event counts and proportions across all demographic and clinical groups are detailed in Table 1. Furthermore, absolute cumulative milestones are provided in Figure 1 and Section 3.2, explicitly highlighting that 50% of recovering patients achieved remission within 18 months and 92.58% achieved it within a critical 24-month window. Longitudinal absolute probabilities are visually represented using stratified Kaplan-Meier curves across the 60-month follow-up period (Figures 3 and 4).

Other analyses	17	Report other analyses done—eg analyses of subgroups and interactions, and sensitivity analyses	**Page No.**: Results Section (Figures 2, 3, 4, 5)	KM curves showing stratified survival/remission rates by hospital, gender, comorbidity, and family history
**Discussion**				
Key results	18	Summarise key results with reference to study objectives	**Page No. / Section**: Discussion Section	Discussion This retrospective cohort study investigated the time to first remission and associated factors in a cohort of 1162 Type 2 Diabetes Mellitus (T2DM) patients treated at two referral hospitals in Eastern Ethiopia. Our findings revealed a dynamic pattern of remission, with a substantial proportion of patients achieving remission within the first two years of treatment, followed by a significant decline in the remission rate. Gender, comorbidities, age, family history of diabetes, cholesterol levels, and the treating hospital had a substantial impact on the time to first remission, emphasizing the importance of a nuanced and tailored approach to T2DM care in this study setting
Limitations	19	Discuss limitations of the study, taking into account sources of potential bias or imprecision. Discuss both direction and magnitude of any potential bias	**Page No. / Section Name:** Discussion Section	However, this study has limitations that must be noted. As a retrospective study, it has the possibility of data inconsistencies and missing information. The study only included patients from two hospitals, so it may not be representative of the entire T2DM community in Eastern Ethiopia. The relatively short follow-up time and censored nature of the study may have reduced the ability to observe long-term effects and estimate the durability of remission.
Interpretation	20	Give a cautious overall interpretation of results considering objectives, limitations, multiplicity of analyses, results from similar studies, and other relevant evidence	**Page No. / Location:** Discussion Section	Contrary to the traditional understanding of age as a risk factor for disease progression, our findings indicated that older age was associated with a shorter time to first remission[28]. This apparent paradox may be explained by higher levels of treatment adherence and health-seeking behavior often observed in older patient populations within this region. Younger patients may face unique barriers, such as occupational demands or lower perceived risk, leading to inconsistent follow-up[29]. Furthermore, this trend may reflect a 'survivor bias,' where older individuals in the cohort represent a subset with fewer life-threatening comorbidities or more stable metabolic profiles, allowing for a quicker response to therapeutic interventions compared to younger counterparts who may present with more aggressive or early-onset forms of the disease[28].<br>One of the key findings was the association between male gender and a longer time to first remission. This observation, which is consistent with certain earlier investigations [11], [22]–[24], necessitates further investigation to determine the underlying mechanisms. The possible explanations One of the key findings was the association between male gender and a longer time to first remission. This observation, which is consistent with certain earlier investigations [11], [22]–[24], necessitates further investigation to determine the underlying mechanisms. The possible explanations could be gender-specific differences in illness etiology,

				therapeutic response, and adherence to treatment regimens[8], [16], [22], [30], [31]. It implies that gender may influence illness progression, therapy response, and treatment regimen adherence.<br>Type 2 Diabetes Mellitus (T2DM) patients with comorbidities had a significantly longer duration to first remission than those without comorbidities. The large impact of comorbidities on the time to first remission is consistent with previous research findings[32], [33]. The presence of other chronic illnesses might complicate diabetes management and increase the risk of complications, thereby prolonging the time required to achieve and sustain remission[34].<br>Our findings also highlight the influence of age and family history of diabetes on remission time. Older age and a positive family member history of diabetes were related to shorter remission times, most likely due to the cumulative influence of the disease and genetic susceptibility, respectively [2], [8], [10], [19], [32], [34]–[36].<br>Another finding of the present study was a substantial difference in remission time between patients treated in the two facilities patients at Hiwot Fana University Specialist Hospital had remission much faster than those at Dil-Chora Referral Hospital. Variations in access Another finding of the present study was a substantial difference in remission time between patients treated in the two facilities patients at Hiwot Fana University Specialist Hospital had remission much faster than those at Dil-Chora Referral Hospital. Variations in access to specialized care, disparities in treatment protocols and adherence, resource availability, and variations in the patient groups treated by each hospital could all contribute to this disparity[37], [38]. Further investigation is warranted to identify the specific factors contributing to this disparity and to explore strategies for improving outcomes at Dil-Chora Referral Hospital.
Generalisability	21	Discuss the generalisability (external validity) of the study results	**Section**: Discussion Section	The paper discusses applicability to other multi-center referral facilities in eastern Ethiopia. However, the study only included patients from two hospitals, so it may not be representative of the entire T2DM community in Ethiopia.
**Other information**				
Funding	22	Give the source of funding and the role of the funders for the present study and, if applicable, for the original study on which the present article is based	Under the Declarations section	No funding was received except facilitation support from Dire Dawa University for the data collection.
